# Beyond Dural Puncture: A Retrospective Analysis of Obstetric Post-spinal Headaches at a Tertiary Centre in the UAE

**DOI:** 10.7759/cureus.108292

**Published:** 2026-05-05

**Authors:** Sneha Celine Jossie, Uday Ambi, Mahima Gajanan Kamath, Salman Muhammad Soomar

**Affiliations:** 1 Internal Medicine, King’s College Hospital London, Dubai, ARE; 2 Anesthesiology, King’s College Hospital London, Dubai, ARE; 3 Pediatrics, King’s College Hospital London, Dubai, ARE; 4 Public Health, King’s College Hospital London, Dubai, ARE

**Keywords:** anesthesia, catheters, dural puncture, epidural, obstetrical, post-dural puncture headache, quality improvement

## Abstract

Background

Post-dural puncture headache (PDPH) remains the most frequent complication of neuraxial anaesthesia, particularly following accidental dural puncture (ADP). Although often self-limiting, PDPH contributes to significant morbidity and prolonged inpatient stay, increasing medico-legal risk. Recent guidelines emphasise structured documentation, early recognition, and preventive measures such as intrathecal catheter (ITC) placement. This audit aimed to evaluate the incidence, risk factors, management patterns, and outcomes of PDPH at a tertiary obstetric unit over six years, with a specific focus on the effectiveness of intrathecal catheterisation following ADP.

Methods

Institutional records were reviewed for all patients diagnosed with PDPH between 2019 and 2025. Data collected included demographics, anaesthetic techniques, ADP incidence, management approach, and outcomes. Descriptive statistics were used to summarise findings and any trends that may have changed over time.

Results

Among 3,594 spinal epidural procedures, 63 cases of PDPH were identified (1.75%). ADP occurred in 15 patients (0.42%), of whom 10 (66.7%) received ITC placement. Headache developed in 2 of 10 patients with a catheter (5%), compared with four of five without (10.3%). PDPH incidence rose from 0.03% in 2019 to 0.61% in 2023, then declined to 0.28% in 2025.

Conclusion

PDPH incidence and ADP rates in this institution were comparable to international data. Intrathecal catheterisation following ADP was associated with a lower likelihood of headache. The findings support the continued use of preventive strategies, structured documentation, and early recognition protocols to optimise PDPH management and outcomes.

## Introduction

Post-dural puncture headache (PDPH) is a common complication after the administration of neuraxial anaesthesia, especially following spinal procedures or accidental dural puncture (ADP) during epidurals [1]. It is generally described as the onset of a positional headache that improves when supine, typically within five days of the puncture, and often accompanies neck stiffness, alongside auditory or visual symptoms [1]. Although usually self-limiting, PDPH may prolong hospital stay, delay maternal-infant bonding, prompt readmission, and lead to medicolegal issues, making it clinically and organisationally significant [1].

The incidence of PDPH varies among patients, techniques, and needle types. It is reported at 0.5%-2% after spinal anaesthesia with small-gauge non-cutting needles but rises to 50%-80% following ADP with a Tuohy epidural needle [2,3]. A five-year obstetric audit found ADP rates of approximately 1%-2%, with most cases developing PDPH [2]. In contrast, a nine-year audit demonstrated a declining incidence following the introduction of atraumatic needles and structured operator training [3]. Similarly, a Canadian 10-year review identified patient- and procedure-related risk factors and confirmed the effectiveness of epidural blood patch (EBP) in the management of PDPH [4].

Regional data are scarce, but a four-year audit from a tertiary maternity hospital in Qatar (2017-2020) reported ADP and PDPH rates comparable to international figures, with EBP as the main treatment modality [5]. This highlights that PDPH remains a significant clinical concern across healthcare systems, including those in the Gulf region.

Treatment approaches range from conservative measures - including bed rest, hydration, caffeine, and simple analgesia - to invasive interventions. EBP remains the gold standard for severe or persistent PDPH [4]. Although EBP has a reported success rate exceeding 90%, some patients may still experience incomplete relief or go on to develop chronic headache syndromes [4]. A recent multicentre meta-analysis demonstrated that intrathecal catheter (ITC) placement following recognised ADP significantly reduced PDPH incidence and the subsequent need for EBP, supporting its role as an evidence-based preventive strategy [6].

Emerging evidence challenges the view of PDPH as a short-lived complication. A nine-year obstetric audit found that headaches persisted beyond the postpartum period in a subset of patients [7], and a large multicentre cohort reported a significant association between severe PDPH and the later development of chronic headache and back pain [8]. Beyond individual morbidity, institutional audits have repeatedly revealed gaps in care - including incomplete documentation, inconsistent patient counselling, and limited structured follow-up - highlighting the need for systematic approaches to improving care quality [2,3,5].

In 2023, the Regional Anesthesia and Pain Medicine (RAPM) consensus guideline introduced a framework for improving the management of both ADP and PDPH [1]. Key recommendations included standardised documentation of ADP, early recognition of PDPH symptoms, prompt administration of EBP, and consideration of ITC placement as part of best clinical practice [1]. These guidelines provide clear benchmarks against which local practice can be evaluated and improved.

The clinical importance of PDPH, advances in ITC placement, and ongoing challenges with documentation and follow-up collectively underscore the need for a local practice assessment. This audit evaluates the incidence, risk factors, management strategies, and outcomes of PDPH at our institution over six years, with a particular focus on the role of ITC use following ADP. Our findings aim to identify practice gaps, guide targeted interventions, and improve patient care in alignment with current international guidelines.

Aim and objectives

The primary aim of this audit was to evaluate the incidence, risk factors, and management outcomes of PDPH following spinal anaesthesia over six years. The objectives were to determine the incidence of PDPH, identify patient- and procedure-related risk factors, examine the management strategies used (conservative versus invasive), and compare outcomes with international standards.

Our audit also had a secondary aim: to assess the role and effectiveness of ITC placement in the management of ADP. This broadens our study, allowing us to explore preventive strategies and inform quality improvement initiatives. The ultimate goal is to optimise patient care and align local practice with best-practice guidelines, making this secondary aim essential to achieving that goal.

## Materials and methods

This was a retrospective, single-centre audit conducted at King’s College Hospital London - Dubai, a tertiary obstetric referral unit in the United Arab Emirates, over a six-year period from January 2019 to December 2025. Ethical approval was granted by the institutional ethics committee (Reference: KCH-2025-097).

Study population and sampling

All obstetric patients who underwent neuraxial anaesthesia during the study period were considered the source population. From these, all patients with a confirmed diagnosis of PDPH or documented ADP were identified through electronic health records and included using a consecutive sampling approach. No sample size calculation was performed as this represented a complete census of all eligible cases within the defined time frame.

Inclusion and exclusion criteria

Obstetric patients with a confirmed diagnosis of PDPH or documented ADP between January 2019 and December 2025, with complete electronic health records, including demographic, procedural, and outcome variables.

Patients with incomplete medical records, and those with PDPH arising from non-obstetric procedures such as diagnostic lumbar puncture.

Data collection

Data were extracted using a structured data collection sheet and included patient demographics (age, body mass index (BMI), and mode of delivery); type of neuraxial procedure; needle gauge; number of insertion attempts; operator details; occurrence and timing of ADP; clinical features of PDPH; management strategies (conservative treatment, EBP, ITC, or sphenopalatine ganglion block (SGBP)); duration of symptoms; and length of hospital stay. Management decisions were made at the discretion of the treating consultant anaesthetist. Conservative management was initiated as first-line treatment in all cases, comprising bed rest, oral hydration, caffeine, and simple analgesia. EBP was offered to patients with severe or persistent symptoms not responding to conservative measures. SGBP was considered as an alternative where EBP was declined or contraindicated. Local practice was benchmarked against the 2023 RAPM consensus guidelines [1], with standards including structured documentation of ADP, early identification of PDPH, timely administration of EBP, and consideration of ITC placement as part of best clinical practice. 

Confounding factors

Potential confounders included variation in operator experience, differences in needle gauge and technique, and changes in documentation practices over time. These were not formally controlled due to the retrospective design.

Statistical analysis

Continuous variables such as age were summarised using mean and standard deviation (SD). Non-normally distributed variables (needle gauge, number of attempts, symptom duration, and hospital stay) were reported as median with interquartile range (IQR). Categorical variables were expressed as frequencies and percentages. Year-wise trends in PDPH incidence from 2019 to 2025 were evaluated and presented as a line chart. All analyses were descriptive; no inferential statistical testing was performed.

## Results

**Figure 1:**
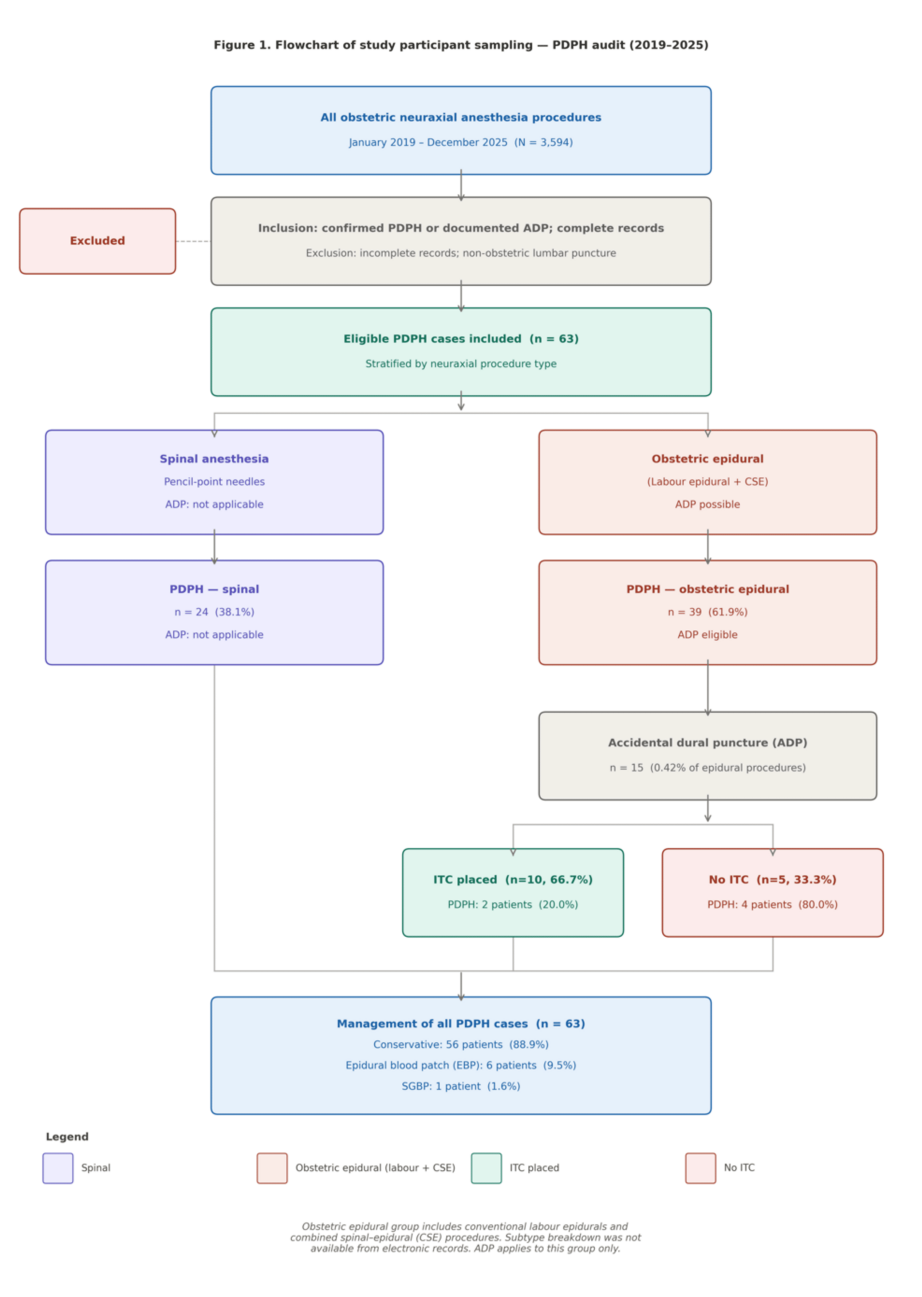
Flowchart of study participant identification and sampling PDPH: post-dural puncture headache; ADP: accidental dural puncture; ITC: intrathecal catheter; CSE: combined spinal-epidural; EBP: epidural blood patch; SGBP: sphenopalatine ganglion block

A total of 3,594 obstetric neuraxial procedures were performed during the study period, from which 63 patients with PDPH were identified, giving an overall incidence of 1.75%. Of these, 15 cases (23.8%) occurred in the setting of ADP, while the remaining 48 (76.2%) arose following intentional dural puncture during spinal anaesthesia or without documented ADP during epidural procedures (Figure 1). 

The mean ± standard deviation (SD) age was 34.9 ± 5.0 years. Baseline and clinical characteristics of all 63 patients are summarised in Table 1. Notably, the majority of patients were in the overweight or obese category, and labour epidural was the most common anaesthetic technique. The median needle gauge was 25 (IQR 18-27), the median number of insertion attempts was one (IQR 1-3), the median symptom duration was two days (IQR 1-3), and the median length of hospital stay was three days (IQR 2-4). All anaesthetic procedures were performed by anaesthetists with a minimum of 15 years of post-residency experience.

**Table 1:** Baseline and clinical characteristics of patients with headache (n = 63) NVD: normal vaginal delivery; CS: caesarean section; EBP: epidural blood patch; SGBP: sphenopalatine ganglion block

Characteristic	n %
Age (Mean ± SD)	34.85 ± 5.02
BMI	
Underweight	1 (1.59)
Normal weight	19 (30.16)
Overweight	30 (47.62)
Obesity	13 (20.63)
Mode of Delivery	
NVD	34 (53.97)
CS	29 (46.03)
Anaesthesia	
Spinal	24 (38.10)
Epidural	39 (61.90)
Needle Gauge	
Median (IQR)	25 (18-27)
Attempts (numbers)	
Median (IQR)	1 (1-3)
Accidental Dural Puncture	
Yes	15 (23.81)
No	48 (76.19)
Duration of Symptoms (days)	
Median (IQR)	2 (1-3)
Mode of Management	
Conservative	56 (88.89)
EBP	6 (9.52)
SGBP	1 (1.59)
Length of Hospital Stay (days)	
Median (IQR)	3 (2-4)

ADP occurred in 15 patients (0.42% of all neuraxial procedures), and the management and headache outcomes of these cases are summarised in Table 2. Of the 15 ADP patients, 10 (66.7%) received an ITC, and five (33.3%) did not. Headache developed in 2 of 10 ADP patients who received an ITC (20.0%), compared with four of five patients without a catheter (80.0%), suggesting a potential protective effect of intrathecal catheterisation.

**Table 2:** Incidence rate of headache

Characteristic (n = 3594 neuraxial procedures)	n %
Incidence of accidental dural puncture (ADP)	15 (0.42)
Cases of ADP received an intrathecal catheter (ITC) (n = 15)	
Yes	10 (66.67)
No	5 (33.33)
Incidence of headache in ADP cases (n = 15)	
With catheter	2 (20.0)
Without catheter	4 (80.0)

Regarding management, 56 patients (88.9%) were treated conservatively, six (9.5%) received an EBP, and one (1.6%) underwent an SGBP.

**Figure 2:**
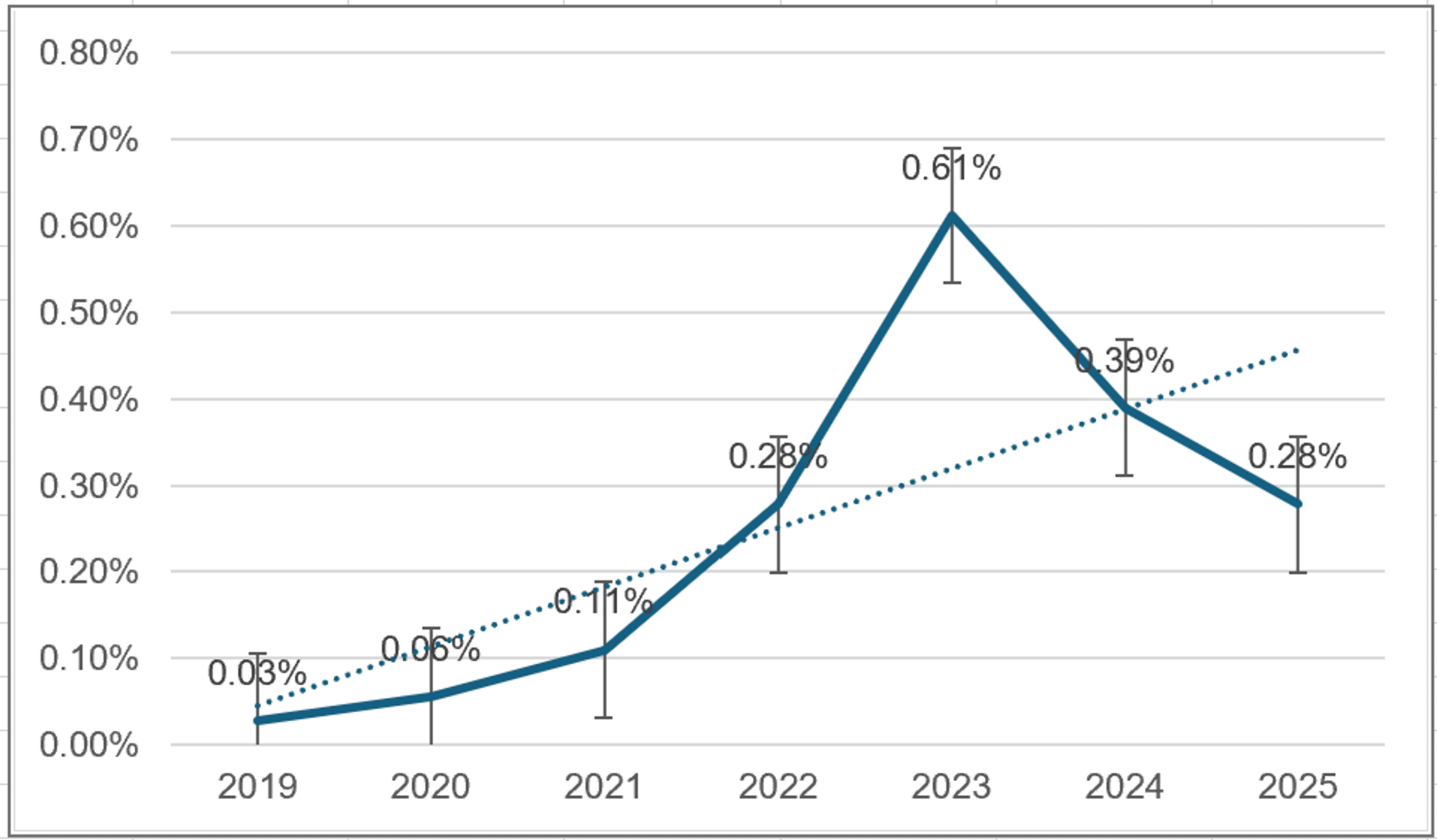
Year-wise incidence of PDPH (2019-2025) The dotted line represents the overall trend in PDPH incidence over the study period. PDPH: post-dural puncture headache

Departmental data showed a gradual rise in headache incidence from 0.03% in 2019 to a peak of 0.61% in 2023, followed by a decline to 0.28% in 2025, as illustrated in Figure 2.

The overall pattern shows a modest upward trend over the audit period, peaking in 2023, followed by a slight decline thereafter.

## Discussion

This audit analysed six years of institutional data on PDPH and ADP, providing insight into local incidence, risk factors, and management patterns in obstetric anaesthesia. The overall PDPH incidence of 1.75% and ADP incidence of 0.42% are comparable to rates previously recorded in tertiary-care audits [2,8].

A nine-year audit conducted in Singapore reported a decline in PDPH incidence following the implementation of atraumatic needles and enhanced operator training [3]. In contrast, our study indicated a gradual increase in PDPH incidence over the six-year period, peaking in 2023 before a subsequent decline. The rising PDPH incidence observed between 2019 and 2023 is likely multifactorial. While improved documentation practices and heightened departmental awareness may have contributed to more complete case identification, rather than a true rise in complication rates, other factors cannot be excluded. These include increased procedural complexity; changes in patient demographics - notably the high proportion of overweight and obese patients in this cohort (68%); and potential variations in needle technique. The subsequent decline from 2023 onward likely reflects a combination of factors, including standardised reporting and procedural refinements, rather than operator experience alone. All anaesthesia providers in our department have at least 15 years of experience, which is consistent with the well-established association between operator seniority and reduced ADP rates [3,4].

Consistent with recent evidence [6,9], ITC placement following ADP was associated with lower headache incidence and a reduced need for an EBP. Recent guidelines from the Obstetric Anaesthetists' Association acknowledge the potential role of ITC placement following recognised ADP, while emphasising that its use should be individualised rather than routine [10]. Within this cohort, 66.7% of ADP cases underwent catheterisation, with favourable outcomes compared with those managed without catheterisation. While EBP remains the gold standard for severe or refractory PDPH [4], its relatively low utilisation rate of 9.5% in this cohort may reflect the effectiveness of preventive strategies, including intrathecal catheterisation and conservative management in selected patients.

A nine-year multicentre cohort and a large prospective audit have both highlighted the potential for persistent headache and back pain extending beyond the immediate postpartum period [7,8]. These findings reinforce the need for structured follow-up and robust documentation practices, as incomplete records and inconsistent patient counselling continue to limit accurate incidence reporting across institutions [2,5].

Assessment of documentation practices at our institution revealed that, prior to this audit, no standardised template existed for recording ADP events, patient counselling, or postpartum follow-up in PDPH cases. This lack of structured documentation likely contributed to incomplete data capture and may have resulted in underreporting of PDPH incidence during the earlier years of the audit period. Following completion of this audit, a dedicated PDPH-specific documentation template incorporating structured counselling records was introduced, representing a direct and tangible quality-improvement outcome of this review.

Based on these findings, several quality-improvement initiatives are recommended. A standardised PDPH documentation template has already been introduced at our institution following this audit, addressing a previously identified gap in record-keeping [1]. This template incorporates structured fields for recording ADP events, symptom onset and severity, patient counselling, and postpartum follow-up, ensuring that future cases are consistently and comprehensively documented. Building on this, a structured postpartum follow-up system should be implemented to capture delayed or persistent symptoms [7,8]. Mandatory reporting of all ADP events should be enforced to facilitate accurate audit data and institutional benchmarking [1,5]. Finally, regular simulation-based epidural training is recommended to improve procedural proficiency and reduce complication rates [3]. A re-audit following full implementation of these measures will be essential to evaluate their long-term impact on clinical outcomes.

The adoption of these quality-improvement measures is expected to improve early recognition and management of PDPH, reduce the incidence of chronic sequelae, enhance patient satisfaction, and ensure compliance with the 2023 RAPM consensus guidelines [1]. A re-audit following implementation will be essential to evaluate the sustainability of these changes and their long-term impact on clinical outcomes.

This study has several notable strengths. It represents one of the largest single-centre retrospective audits of PDPH and ADP conducted over an extended six-year period, providing a comprehensive longitudinal overview of incidence trends and management patterns. Furthermore, given the scarcity of published data from the Gulf region, this study makes a valuable contribution to the regional literature and offers a benchmark for other institutions in the United Arab Emirates and wider Gulf Cooperation Council countries to evaluate their own practice against international standards.

Despite these findings, several limitations must be acknowledged. The retrospective design relied on existing medical records, which may have introduced selection bias and limited data completeness. As a single-centre analysis from one institution in the United Arab Emirates, the findings may not be fully generalisable to other settings. A further limitation is the inability to report the exact breakdown of procedure subtypes (spinal, epidural, and CSE) across all 3,594 procedures, as this level of detail was not consistently recorded in the electronic health records. As a result, procedure-specific PDPH incidence rates could not be calculated. The relatively small number of ADP cases limits the power to draw definitive conclusions regarding the protective effect of ITC placement, and a prospective study with a larger sample would be required to confirm this association. Nevertheless, given the scarcity of published data from the Gulf region, this study provides valuable regional benchmarking that may inform local clinical practice and serve as a foundation for future multicentre research.

## Conclusions

This six-year retrospective analysis demonstrates that the incidence of PDPH and ADP at our institution is comparable with international benchmarks. The observed association between ITC placement and lower headache incidence highlights an opportunity to strengthen preventive strategies following ADP. The implementation of standardised documentation, structured postpartum follow-up, and targeted training initiatives may support sustained quality improvement in the recognition, management, and patient outcomes.
